# Prevalence and association of frailty with SARS-CoV-2 infection in older adults in Southern Switzerland—Findings from the Corona Immunitas Ticino Study

**DOI:** 10.1186/s12877-023-03730-7

**Published:** 2023-01-12

**Authors:** Miao Jiang, Laurie Corna, Rebecca Amati, Giovanni Piumatti, Giovanni Franscella, Luca Crivelli, Emiliano Albanese

**Affiliations:** 1 Institute of Public Health, Università della Svizzera italiana, Lugano, Switzerland; 2 Department of Business Economics, Health and Social Care, University of Applied Sciences and Arts of Southern Switzerland, Manno, Switzerland; 3 Fondazione Agnelli, Turin, Italy; 4 Department of Psychiatry, University of Geneva, Geneva, Switzerland

**Keywords:** Frailty, Epidemiology, Older adults, COVID-19

## Abstract

**Background:**

Frailty is an age-associated state of increased vulnerability to stressors that strongly predicts poor health outcomes. Epidemiological evidence on frailty is limited during the COVID-19 pandemic, and whether frailty is associated with the risk of infection is unknown.

**Objectives:**

We derived a robust Frailty Index (FI) to measure the prevalence of frailty and its risk factors in community-dwelling older adults in Southern Switzerland (Ticino), and we explored the association between frailty and serologically confirmed SARS-CoV-2 infection.

**Methods:**

In September 2020, we recruited a random sample of community-dwelling older adults (65 +) in the Corona Immunitas Ticino prospective cohort study (CIT) and assessed a variety of lifestyle and health characteristics. We selected 30 health-related variables, computed the Rockwood FI, and applied standard thresholds for robust (FI < 0.1), pre-frail (0.1 ≤ FI < 0.21), and frail (FI ≥ 0.21).

**Results:**

Complete data for the FI was available for 660 older adults. The FI score ranged between zero (no frailty) and 0.59. The prevalence of frailty and pre-frailty were 10.3% and 48.2% respectively. The log-transformed FI score increased by age similarly in males and females, on average by 2.8% (*p* < 0.001) per one-year increase in age. Out of 481 participants with a valid serological test, 11.2% were seropositive to either anti-SARS-CoV-2 IgA or IgG. The frailty status and seropositivity were not statistically associated (*p* = 0.236).

**Conclusion:**

Advanced age increases the risk of frailty. The risk of COVID-19 infection in older adults may not differ by frailty status.

**Supplementary Information:**

The online version contains supplementary material available at 10.1186/s12877-023-03730-7.

## Introduction

The world’s population aged 65 years or older is expected to more than double from 0.7 billion in 2019 to 1.5 billion in 2050 [1]. Though global population ageing is a major societal achievement, it poses enormous public health challenges. Screening and monitoring age-related health conditions such as frailty is crucial [2].

Frailty is a geriatric condition characterized by increased vulnerability to endogenous and exogenous stressors, which is strongly associated with dependency and increased mortality [3, 4]. However, the construct validity of frailty is still debated, and consensus is lacking on how it should be assessed [2]. The Rockwood´s Frailty Index (FI) is a highly adaptive approach whereby frailty is conceived as an accumulation of health deficits that span a variety of health-related domains [5]. The FI approach has become increasingly popular because it is a better predictor of health-related adverse consequences than other frailty measurements [4, 6]. Moreover, the FI approach does not necessarily require a physical examination and it can be derived from pre-existing electronic health records or data from health surveys [4, 7, 8].

Previous studies showed that frailty might contribute to dysregulations of the immune system including low grade chronic inflammation, altered general and specialized immune response [9], which may contribute to the susceptibility to COVID-19 infection [10]. During the COVID-19 pandemic, most studies related to frailty were conducted in clinical settings typically using Clinical Frailty Scale [11], often administered by experienced health professionals [12]. Evidence suggests that frailty is a strong predictor of developing severe forms of COVID-19 and is associated with COVID related death in clinical settings [13–15]. However, evidence is limited on the prevalence of frailty in the general population during the COVID-19 pandemic, and whether frailty is associated with SARS-CoV-2 infection in community dwelling older adults.

We derived a robust FI combining a wide array of measures from an existing population-based study on the impact of the COVID-19 pandemic. We aimed to 1) calculate the prevalence of frailty in community-dwelling older adults in Southern Switzerland (Ticino); 2) explore the risk factors of frailty; and 3) explore the association between frailty and serologically confirmed COVID-19 infections.

## Methods

### Study design

Corona Immunitas Ticino (CIT) is a population-based prospective seroprevalence study that started in July 2020, after the first COVID-19 epidemic wave and lockdown in Southern Switzerland. The main aim of CIT was to assess the spread and impact of the COVID-19 pandemic. CIT is part of Corona Immunitas (CI), a nationwide research program led by the Swiss School of Public Health (SSPH +) [16]. Detailed information about the study design is reported elsewhere [16, 17].

### Study setting and participants

CIT was conducted in Southern Switzerland—Canton Ticino, which borders Lombardy, Italy, the epicentre of the epidemic in Europe. We recruited participants using age- and sex- stratified random sampling based on regional registries of the Federal Office of Statistics. For this study on frailty, we considered 874 older adults aged 65 years and older, who agreed to participate in September and November 2020, provided informed consent and responded to the baseline questionnaire. Of these, we excluded 114 participants due to missing values for the computation of the FI (described below). This reduced the analytical sample size (n1) for the FI construction to 660. The included and excluded participants had similar age distributions. For the further assessment of the relationship between frailty and seroprevalence, we analysed data of the subsample of older adults who took part in the serosurvey and who had a valid serological test (n2 = 481).

### Data collection

The Research Electronic Data Capture (REDCap) [18, 19], a secure, web-based platform hosted at the Università della Svizzera italiana (USI) was used for questionnaire development, data collection, storage, and management. Older participants were also offered the possibility to participate over the phone with a dedicated interviewer using computer-assisted telephone interviewing (CATI).

We asked participants to complete a baseline questionnaire following registration with the study. One week after the completion of the baseline assessment, we administered monthly and weekly questionnaires for repeated measures. We enquired about socio-demographic characteristics, physical and psychological health status, social relationships, and lifestyles of participants and their household environments.

To measure seropositivity to SARS-CoV-2, we invited participants to two rounds of blood testing. Professional nurses collected peripheral venous blood samples at a chosen healthcare facility or in their homes. The samples were analysed with the Luminex binding assay SenASTrIS (Sensitive Anti-SARS-CoV-2 Spike Trimer Immunoglobulin Serological) to detect SARS-CoV-2 antibodies. Previous validations in population-based samples showed high sensitivity and specificity [20].

For the current paper, we used data from the baseline questionnaire, the third monthly questionnaire and the laboratory results from the serological test performed between November 2020 and January 2021 (before the introduction of vaccines).

### Frailty Index Construction

We derived the Rockwood FI using the method described by Searle et al. [4]. We included 30 variables that covered seven domains including chronic diseases, basic activities of daily living, instrumental activities of daily livings, lifestyle, physical measurements, self-reported health status, and psychological symptoms (Supplementary Table 1). The rationale of the FI is that the deficits/impairments should be related to ill-health status, should cover a variety of health domains, should progressively increase with age, and should not saturate at relatively younger age. The six deficits related to psychological symptoms and signs were selected from the third monthly questionnaire and the remaining 24 deficits were extracted from the baseline questionnaire. The FI is calculated as the ratio of the sum of deficits reported to the total number of deficits considered, and we applied a previously validated cut-off (i.e., 0.21) for frailty caseness [21]. We considered 0.1 ≤ FI < 0.21 as pre-frail, FI < 0.1 as robust [21]. For example, if a participant reported 6 out of 30 deficits considered, his/her FI would be 0.2, and would be classified as pre-frail. Before computing the FI, we recoded binary variables according to their possible answers as either ‘0’ or ‘1’, with ‘1’ representing the presence of a health deficit. For ordinal variables, we used a Likert-like scale. We assigned values of ‘0’, ‘0.5’, ‘1’ to variables with 3 possible answers; ‘0’, ‘0.33’, ‘0.67’, ‘1’ to variables with 4 possible answers; and ‘0’, ‘0.25’, ‘0.5’, ‘0.75’, ‘1’ to variables with 5 possible answers. For the continuous variables, such as BMI (Body Mass Index), we used universally agreed cut-offs [22] to categorize the responses (see Supplementary Table 1), then we assigned values accordingly using the abovementioned method.

### Covariates

For the analysis of potential risk and protective factors of frailty, we used sociodemographic and lifestyle measures collected at baseline including age (modelled as continuous and categorical variable for analytic purposes, namely 65–69 years old, 70–74 years old, 75–79 years old and 80 years old and plus); sex (male/female), income satisfaction (i.e. ‘not enough’, ‘enough’ and ‘more than enough’), education (‘none’, ‘compulsory education’, ‘higher secondary education’, and ‘university’) and smoking (‘non-smoker’, ‘past smoker’ and ‘current smoker’).

To explore whether there is any relationship between frailty and serologically confirmed SARS-CoV-2 infection, we included in our models the abovementioned sociodemographic factors, multimorbidity and frailty status (robust, pre-frail, frail). The multimorbidity was operationalized as a binary variable (yes/no). Participants who reported at least two chronic conditions were considered having multimorbidity: cardiovascular diseases (e.g., angina pectoris, peripheral vascular diseases, intermittent claudication, heart attack, stroke or heart failure), chronic respiratory conditions (chronic obstructive pulmonary disease, chronic bronchitis, emphysema or asthma), allergy (pollen allergy or hay fever), immune disorders, hypertension, diabetes mellitus, cancer or other conditions.

### Statistical analysis

We used complete case analysis in our study because those participants with missing values on the items used to construct the FI were not significantly different from those with no missing values across socio-demographic characteristics. We first describe the socio-demographic characteristics of the sample. Means and standard deviation (SD) were used for normally distributed data, median and interquartile range (IQR) for skewed data, and Spearman correlations and Wilcoxon rank sum tests to compare groups.

We used Poisson regression to estimate the prevalence of frailty by sex and age group. We used log-transformed linear regressions to explore the association between age and FI. In addition, we used univariate and multivariable multinomial logistic regressions to assess associations between age group, sex, income satisfaction, education level and smoking status and frailty status.

Potential risk factors for SARS-CoV-2 seropositivity including age group, sex, smoking status, multimorbidity and frailty status, were tested separately using logistic regressions. A *p* value less than 0.05 was considered statistically significant. We performed all analyses using Stata Version 17.0 [23].

### Sensitivity analysis

We conducted two sets of sensitivity analysis. Firstly, we used two-sample Kolmogorov–Smirnov test and the Mann–Whitney U test to compare the distribution of FI and the mean score of FI between the analytical sample who participated in the serological blood sampling and the sample who did not. We assumed that frail older people might be less likely to participate in the serological blood sampling which may contribute to dilute the results of the potential relationship between frailty and COVID-19 infection.

Secondly, we explored whether there is any potential relationship between sub-components of frailty and COVID-19 seropositivity using logistic regression. The seven subcomponents of the FI and the sum of all frailty components were included individually to the model as independent variables. The chronic diseases component’s score ranged from zero to eight, the one about basic activities of daily livings ranged from zero to five, instrumental activities of daily livings from zero to eight, and psychological symptoms and signs ranged from zero to six. The other three components (self-reported health, physical measurement, and lifestyle) all ranged from zero to one. The overall sum of the frailty items ranged from zero to 30.

## Results

Out of 874 respondents aged 65 years and over, complete data for the FI calculation was available for 660 participants (75.4%). The mean age of the study sample was 73 years, 57% were female, and 77% completed at least higher secondary education. Nearly 40% were either current or past smokers (Table 1). Overall, we identified 68 frail, 318 pre-frail, and 274 robust older adults.

**Table 1:** Sociodemographic of the study sample stratified by frailty level

		**Frailty status**		
	Overall	Robust	Pre-frail	Frail
***n* (%)**	660 (100)	274 (41.5)	318 (48.2)	68 (10.3)
**Age (years ± SD)**	72.7 (5.7)	71.4 (5.0)	73.0 (5.7)	76.2 (7.2)
**Age groups (years)**				
*65–69*	237 (35.9)	124 (52.3)	98 (41.4)	15 (6.3)
*70–74*	212 (32.1)	82 (38.7)	113 (53.3)	17 (8.0)
*75–79*	126 (19.1)	47 (37.3)	66 (52.4)	13 (10.3)
≥ *80*	85 (12.9)	21 (24.7)	41 (48.2)	23 (27.1)
**Sex**				
*Female*	375 (56.8)	144 (38.4)	193 (51.5)	38 (10.1)
*Male*	285 (43.2)	130 (45.6)	125 (43.9)	30 (10.5)
*Missing*	0			
**Education level**				
*None*	8 (1.2)	2 (25)	6 (75)	0 (0)
*Compulsory education*	78 (11.9)	24 (30.8)	37 (47.4)	17 (21.8)
*Higher secondary education*	449 (68.65)	186 (41.4)	222 (49.4)	41 (9.1)
*University education*	119 (18.2)	60 (50.4)	49 (41.2)	10 (8.4)
*Missing*	6			
**Marital status**				
*Single or not married*	30 (4.6)	9 (30)	16 (53.3)	5 (16.7)
*Married or in a civil union*	439 66.9)	192 (43.7)	211 (48.1)	36 (8.2)
*Widowed*	103 (15.7)	33 (32)	50 (48.5)	20 (19.4)
*Divorced/Separated or in a former civil union*	84 (12.8)	37 (44)	40 (47.6)	7 (8.3)
*Missing*	4			
**Smoking status**				
*Non-smoker*	374 (57.2)	151 (40.4)	186 (49.7)	37 (9.9)
*Past smoker*	217 (33.2)	92 (42.4)	99 (45.6)	26 (12)
*Current smoker*	63 (9.6)	27 (42.9)	32 (50.8)	4 (6.3)
*Missing*	6			
**Self-reported income satisfaction**				
*Not enough*	9 (1.5)	3 (33.3)	3 (33.3)	3 (33.3)
*Enough*	327 (53.1)	120 (36.7)	165 (50.5)	42 (12.8)
*More than enough*	280 (45.5)	132 (47.1)	129 (46.1)	19 (6.8)
*Missing*	44			
**Multimorbidity**				
*No*	484 (73.3)	260 (94.9)	208 (65.4)	16 (23.5)
*Yes*	176 (26.7)	14 (5.1)	110 (34.6)	52 (76.5)

*Notes:* Total percentages may not be 100% due to rounding

### Distribution and prevalence of frailty

**Fig. 1:**
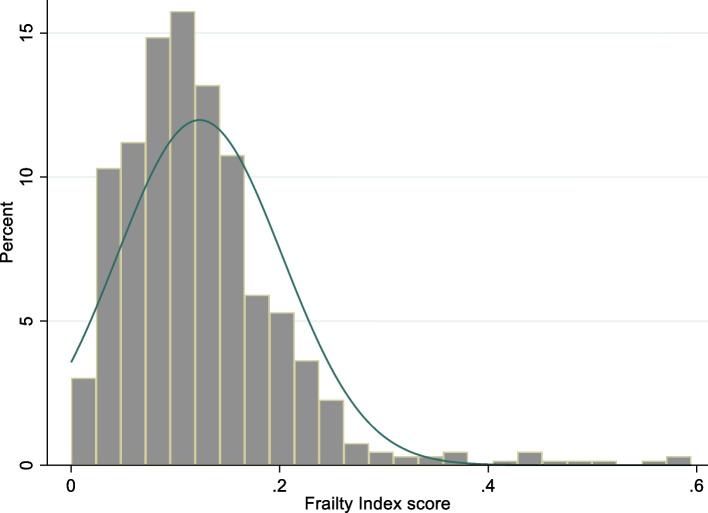
Histogram and density plot of Frailty Index

The distribution of frailty was right skewed (Fig. 1). The FI score ranged from zero to 0.595 with a median of 0.111 (IQR = 0.085), and a mean of 0.123 (SD = 0.079). Table 2 shows the prevalence of frailty stratified by age groups. The prevalence of frailty increased with age (*p* = 0.005) but was similar for men and women at approximately 10%.

**Table 2:** Frailty Index score and frailty prevalence by age group

Age group (N)	Frailty Index score			Frailty prevalence (score ≥ 0.21)		
	Overall Mean (SD)	Female Mean (SD)	Male Mean (SD)	Overall % [95% CI]	Female % [95% CI]	Male % [95% CI]
65–69 (*N* = 237)	0.11 (0.06)	0.11 (0.06)	0.11 (0.07)	6.33 [3.82, 10.50]	5.76 [2.88, 11.51]	7.14 [3.41, 14.98]
70–74 (*N* = 212)	0.12 (0.07)	0.12 (0.07)	0.12 (0.08)	8.02 [4.99, 12.90]	5.17 [2.32, 11.51]	11.46 [6.35, 20.69]
75–79 (*N* = 126)	0.13 (0.07)	0.13 (0.07)	0.13 (0.09)	10.32 [5.99, 17.77]	10.96 [5.48, 21.91]	9.43 [3.93, 22.67]
≥ 80 (*N* = 85)	0.17 (0.11)	0.19 (0.12)	0.15 (0.10)	27.06 [17.98, 40.72]	34.04 [20.86, 55.57]	18.42 [8.78, 38.64]
Total (*N* = 660)	0.12 (0.08)	0.12 (0.08)	0.12 (0.08)	10.30 [8.12, 13.07]	10.13 [7.37, 13.93]	10.53 [7.36, 15.06]

*Notes: SD: standard deviation; CI: confidence interval*

**Fig. 2:**
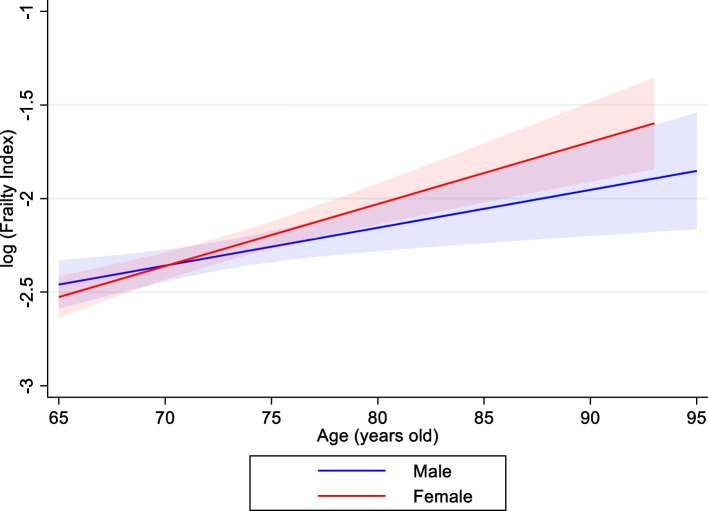
Relationship between age and log-transformed Frailty Index by sex

Participants with higher income satisfaction had a higher median FI score compared to those who reported lower income (Median = 0.103, IQR = 0.082). Median FI scores were also inversely associated with higher education level (*p* < 0.05). Those who did not complete compulsory education had a median FI score of 0.136 (IQR = 0.054), and people who received university education had a lower median score (i.e., were less frail) 0.099 (IQR = 0.080). We found no difference in FI scores by sex (*p* = 0.275) or between smokers and non-smokers (*p* = 0.612). In Fig. 2, the log-transformed FI score increased with age similarly in males and females, on average by 2.8% (SE = 0.004, *p* < 0.001) per one-year increase in age.

In multinomial mutually adjusted models, pre-frailty and frailty were respectively 1.28 and 1.90 times more likely by every 5-year age increments (*p* < 0.05), and higher income satisfaction was related to lower risk of frailty (*p* < 0.05) but not with pre-frailty (Table 3).

**Table 3:** Risk factors for frailty in multinomial logistic regression

**Frailty status**	**Relative risk ratio**	***p* value**	**95% CI**
**Robust**	Base outcome		
**Pre-frailty**			
Age group *(per 5-year group)*	1.28	0.006	1.07–1.52
Sex *(male vs female)*	1.40	0.065	0.98–1.99
Income satisfaction *(per level)*^*a*^	0.79	0.184	0.56–1.12
Education level *(per level)*^*b*^	0.73	0.056	0.53–1.01
Smoking status *(per level)*^*c*^	0.92	0.528	0.71–1.20
**Frailty**			
Age group *(per 5-year group)*	1.90	0.000	1.45–2.50
Sex *(male vs female)*	1.22	0.513	0.67–2.21
Income satisfaction *(per level)*^*a*^	0.44	0.006	0.25–0.79
Education level *(per level)*^*b*^	0.63	0.072	0.38–1.04
Smoking status *(per level)*^*c*^	0.99	0.969	0.64–1.53

*Notes:*

*CI* confidence interval

^a^Income satisfaction: 1 = not enough, 2 = enough, 3 = more than enough

^b^Education level: 1 = none, 2 = compulsory education, 3 = higher secondary education, 4 = university education

^c^Smoking status: 1 = non-smoker, 2 = past smoker, 3 = current smoker

### Frailty and SARS-CoV-2 infection

To test whether frailty was associated with SARS-CoV-2 infection, we further excluded 179 participants who did not perform or lacked a valid serological test result, resulting in sub-sample of 481 participants. We found that 54 participants (11.2%) had developed antibodies against SARS-CoV-2. The proportion of seropositivity increased with frailty status. In the frail group, around 17% had positive serological results compared to 11% in the pre-frail group and 10% in the robust group. The frequencies and proportions of serological results of anti-SARS-CoV-2 antibodies stratified by frailty status are presented in Table 4.

**Table 4:** Serological results of anti-SARS-CoV-2 antibodies stratified by frailty status

	**Overall**	**Non-frail**	**Pre-frail**	**Frail**
**n (%)**	481 (100)	209 (43.45)	226 (46.99)	46 (9.56)
**Serological results (to either IgG or IgA)**				
*Negative*	427 (88.8)	188 (89.95)	201 (88.94)	38 (82.61)
*Positive*	54 (11.2)	21 (10.05)	25 (11.06)	8 (17.39)
**Serological results IgG**				
*Negative*	432 (89.81)	191 (91.39)	204 (90.27)	37 (80.43)
*Positive*	42 (8.73)	16 (7.66)	18 (7.96)	8 (17.39)
*Indetermined*	7 (1.46)	2 (0.96)	4 (1.77)	1 (2.17)
**IgG titres (AU/ml), median (IQR)**	57.2 (29.3, 120.3)	38.8 (16.5, 71.7)	86.2 (40.4, 162.5)	55.6 (37.25, 107.15)
**Serological results IgA**				
*Negative*	439 (91.27)	196 (93.78)	203 (89.82)	40 (86.96)
*Positive*	42 (8.73)	13 (6.22)	23 (10.18)	6 (13.04)
**IgA titres (AU/ml), median (IQR)**	43 (20.4, 104.9)	35.2 (20.4, 87.5)	46.9 (17.3, 101.9)	69.4 (20.7, 347)

*Notes*: *IgG* Immunoglobulin G, *IgA* Immunoglobulin A, *IQR* Interquartile range

The associations between potential risk factors for COVID-19 infection, including age, sex, smoking status, multimorbidity, self-reported health status, and frailty status were not statistically significant. Odds ratios (ORs) for these null associations with 95% robust CIs are presented in Supplementary Table 2.

### Sensitivity analysis

From the results of two-sample Kolmogorov–Smirnov test (*p* = 0.202), we could not reject the null hypothesis that the distributions of FI were the same in those who participated in serological blood sampling and those did not. Moreover, Mann–Whitney U test showed that the mean of FI was not statistically significantly different between these two groups (z = 1.883, *p* = 0.060). In the second set of sensitivity analysis, neither of the seven sub-components of FI nor the overall sum score of frailty item were associated with COVID-19 infection (Supplementary Table 3).

## Discussion

We made use of a large array of measures to construct a 30-item FI in a random and fairly representative sample of older adults participating in an ongoing population-based cohort study during the COVID-19 pandemic. The prevalence of frailty in Southern Switzerland was 10.3% (CI: 8.12, 13.07), and almost half of the study participants were identified as pre-frail. Advanced age and lower income satisfaction were both positively associated with frailty. Neither frailty nor its sub-components was associated with serologically confirmed COVID-19 infection.

Our results on the occurrence of pre-frailty at around 48% are consistent with recent figures from a large meta-analysis that included studies conducted with people aged 50 years and over in 62 countries that found a pre-frailty prevalence of 49% [24]. Conversely, the prevalence of frailty was 24% in the same meta-analysis [24], which is more than twice that found in our study (10%). Moreover, the mean FI score of our sample (0.12) was lower than the mean FI score in Switzerland (0.19) reported in the SHARE study, and in other European countries [25–27]. We collected data during the COVID-19 pandemic, and its associated restrictions may have limited the participation of less healthy individuals, but self-selection of frail individuals in other studies may not be excluded given the different sampling and recruitment procedures between our study and previous studies.

Similar to other studies, the distribution of frailty was right skewed and upper limit was less than 0.7 [4, 26, 28]. It is commonly believed that older women tend to be frailer than men [4, 28–30], however we did not find significant differences by sex. Frailty level increased with age. The growth rate of the log-transformed FI per one year increase in age was 2.8% in our study, which is in line with the 3.5% found in a large Dutch study (LASA) [28]. The frequency of frailty increased with age but slightly decreased in the oldest old. Prevalence bias may not be excluded because frailty may be associated with poorer health and death competing risks in later life. Moreover, in our study the association between frailty and age increased more steeply in women compared to men in contrast to that found for Swiss older adults using data from SHARE [25].

Evidence suggests that older age, low income and multimorbidity may increase the risk of COVID-19 infection, while current smoking may lower it [31–33]. Evidence is inconsistent on the association between frailty and risk of COVID-19 infection. For example, one study showed that frailty was significantly associated with incident COVID-infection (adjusted HR = 7.01, 95% CI = 2.69–18.25) [10]. Consistent with what we observed, frailty was not related to higher risk of COVID-19 infection in a study based on the 11-item Edmonton Frailty Scale [34]. Similarly, a prospective analysis of UK Biobank data did not find associations between frailty (diagnosed using Fried frailty criteria) and multimorbidity with positive PCR test for COVID-19 [35]. Comparisons with previous studies are not straightforward because of the settings, the frailty measurements used, and their construct validity are markedly heterogeneous across studies. Moreover, during the COVID-19 pandemic older people especially those frail ones and those with multimorbidities were recommended to strictly comply with stay-at-home measures. Adherence to the public health measures was high among older population in Switzerland, and higher in those with pre-existing health conditions [36]. Higher adherence to preventive measures likely contributed to reducing contact between infected and susceptible individuals amongst frail and/or multimorbid older adults, counterbalancing the higher duration of infectiousness and probability of infection on contact in this group. Mechanistic evidence suggests that the latter two are higher in frail compared to robust older adults, irrespective of their age [9]. Further large-scale studies using the Rockwood FI approach to assess whether frailty predisposes older adults to COVID-19 infection mitigating the effect of preventive measures are warranted.

### Limitations

Our study has several limitations. Firstly, issues of directionality cannot be addressed in our analysis due to the cross-sectional design, which is, however, robust for the descriptive findings, including estimation of the prevalence of frailty. Moreover, as not all measures were repeated over time, we were unable to explore changes in frailty status through the pandemic waves. We know that frailty is a dynamic health condition [37], and it would be relevant to explore the transition and evolution of frailty status in the long term. Furthermore, although we implemented several measures to facilitate older adults' participation in the study (e.g., CATI and at-home blood sampling), the constraints imposed by the pandemic may have contributed to participation bias favouring healthier individuals. This may have led to an underestimation of the true prevalence of frailty. All the variables used to derive the FI were self-reported, which may have contributed to measurement error and recall bias. Whether this caused an under- or over-estimation of the true prevalence of frailty is unclear. Our results should be generalized with caution to similar populations. Finally, we cannot exclude that we lacked sufficient statistical power to detect a true effect of frailty on COVID-19 infection because of the relatively low seroprevalence in our sample.

### Strengths

Our study provides novel prevalence estimates of frailty based on the operationalization of the FI during the COVID-19 pandemic in Southern Switzerland. Epidemiological evidence on frailty is patchy or outdated in both Western and non-Western countries, due to inherent difficulties in recruiting and assessing frailty in representative samples of older adults.

The FI approach used in this study has several advantages over the traditional Fried frailty phenotype approach [38]. For instance, the phenotype approach is resource intense because it requires in-person interviews and assessments, including hand grip strength with a dynamometer [39]. In comparison, the FI approach relies on indirect indicators of physical fitness inferred from a variety of domains that do not necessarily require a physical examination. This was a main advantage given the physical distancing and hygiene measures and restrictions enforced during the COVID-19 pandemic. An additional advantage of the FI is its multidimensional approach that extends to mental, psychological, and cognitive health [4], all of which were largely impacted during the pandemic. We were able to include 30 health-related measures to construct the FI, a number widely considered sufficient to provide robust frailty estimates and FI scores [4]. It is worth mentioning that the alternative way to treat the ordinal variables is grouping the answers and converting them into dichotomous variables. However, previous research shows that as long as the number of deficits included is sufficient (at least 30), both scoring methods provide nearly identical results in terms of mean FI scores and its ability to predict mortality [40]. The internal validity of our study supports inferential reasoning that may suggest that potentially up to half of the population 65 + of Southern Switzerland could benefit from interventions aimed at preventing and reversing frailty, including dietary supplements or increased intake of vitamin D and proteins [41–43].

## Conclusion

In conclusion, the prevalence of frailty and pre-frailty was high among older adults in Southern Switzerland after the first lockdown and during the COVID-19 pandemic. Our study is exemplar of turning a dreadful public health emergency into an opportunity to collect and use epidemiological data to fill evidence gaps. Prospective data are warranted to monitor changes in frailty occurrence, to study its impact on individuals and society, and to gauge mid- and long-term consequences of the COVID-19 pandemic on frail and pre-frail older adults. However, our findings highlight prevention opportunities of public health significance because of the steady expansion of the older segment of the population in the region and globally.

## Supplementary Information

**Additional file 1: Supplementary Table 1.** Thirty health deficits and their scoring used to derive the Frailty Index. **Supplementary Table 2.** Univariate logistic regression model for potential risk factors of SARS-CoV-2 infection. **Supplementary Table 3.** Univariate logistic regression model to assess the association between sub-components of Frailty Index and SARS-CoV-2 infection—a sensitivity analysis.

## Data Availability

All data sharing and collaboration requests should be directed to the corresponding author MJ (miao.jiang@usi.ch) and the senior author EA (emiliano.albanese@usi.ch).

## Abbreviations

FI: Frailty Index
CIT: Corona Immunitas Ticino
CI: Corona Immunitas
SSPH +: Swiss School of Public Health
REDCap: Research Electronic Data Capture
USI: Università della Svizzera italiana
CATI: Computer Assisted Telephone Interviewing
SenASTrIS: Sensitive Anti-SARS-CoV-2 Spike Trimer Immunoglobulin Serological
IgG: Immunoglobulin G
IgA: Immunoglobulin A
BMI: Body Mass Index
