# Supplementary material for: Prevalence and association of frailty with SARS-CoV-2 infection in older adults in Southern Switzerland—Findings from the Corona Immunitas Ticino Study
Source: BMC Geriatr. 2023 Jan 12;23:18. doi: 10.1186/s12877-023-03730-7 (PMC9834033; doi:10.1186/s12877-023-03730-7)
Supplement: Supplementary file 1 — Additional file 1: Supplementary Table 1. Thirty health deficits and their scoring used to derive the Frailty Index. Supplementary Table 2. Univariate logistic regression model for potential risk factors of SARS-CoV-2 infection. Supplementary Table 3. Univariate logistic regression model to assess the association between sub-components of Frailty Index and SARS-CoV-2 infection—a sensitivity analysis. [file 12877_2023_3730_MOESM1_ESM.docx]

**Supplementary Materials**

**Table 1. Thirty health deficits and their scoring used to derive the Frailty Index.**

| Domains | Number | Variable name | Response/code |
| --- | --- | --- | --- |
| Chronic diseases | 1. | Cancer | No = '0', Yes = '1' |
|  | 2. | Diabetes |  |
|  | 3. | Immunological disorder |  |
|  | 4. | Hypertension |  |
|  | 5. | Cardiovascular diseases |  |
|  | 6. | Respiratory disease |  |
|  | 7. | Allergy |  |
|  | 8. | Other diseases |  |
| Basic activities of daily livings | 9. | Eating without help | Yes, without any difficulty = ‘0’  Yes, but with slight difficulty = ‘0.33’  Yes, but with great difficulty = ‘0.67’  No, I can’t at all = ‘1’ |
|  | 10. | Standing up without help |  |
|  | 11. | Dressing clothes without help |  |
|  | 12 | Going to the toilet without help |  |
|  | 13. | Bathing without help |  |
| Instrumental activities of daily livings | 14. | Cooking without help |  |
|  | 15. | Answering the phone without help |  |
|  | 16. | Shopping without help |  |
|  | 17. | Doing laundry without help |  |
|  | 18. | Doing light household chores without help |  |
|  | 19. | Doing heavy household chores without help |  |
|  | 20. | Managing finance without help |  |
|  | 21. | Taking public transport without help |  |
| Psychological symptoms and signs | 22. | Dry mouth | Never = ‘0’  Sometimes = ‘0.33’ Often = ‘0.67’  Almost always = ‘1’ |
|  | 23. | Not being able to feel positive |  |
|  | 24. | Agitation |  |
|  | 25. | Feeling of lacking companionship | Never = ‘0’  Rare = ‘0.25’ Occasionally = ‘0.5’  Most of the time = ‘0.75’  Always = ‘1’ |
|  | 26. | Feeling of being left out |  |
|  | 27. | Feeling of being isolated from others |  |
| Self-reported health | 28. | Self-reported health status | Very good = '0'  Good = '0.25'  Satisfied/fair = '0.5' Rather bad = '0.75'  Bad = '1' |
| Physical measurement | 29. | Body mass index (BMI) | ≥ 18.5 and < 25 = ‘0’  ≥ 25 and < 30 = ‘0.5’  < 18.5 or ≥ 30 = ‘1’ |
| Lifestyle | 30. | Mild-light physical activities | Yes = ‘0’, No = ‘1’ |

**Table 2. Univariate logistic regression model for potential risk factors of SARS-CoV-2 infection**

|  | **Odds ratio** | ***p* value** | **95% CI** |
| --- | --- | --- | --- |
| Age group *(per 5-year group)* | 1.09 | 0.545 | 0.82–1.44 |
| Sex *(male vs female)* | 0.98 | 0.951 | 0.55­–1.74 |
| Smoking status (per level)^a^ | 0.97 | 0.905 | 0.63–1.50 |
| Income satisfaction (per level)^b^ | 1.62 | 0.095 | 0.92–2.85 |
| Frailty status (per level)^c^ | 1.30 | 0.236 | 0.84–1.99 |
| Multimorbidity (yes vs no) | 1.58 | 0.133 | 0.87–2.88 |

*Notes: ^a^Smoking status: 1=non-smoker, 2=past smoker, 3=current smoker*

*^b^Income satisfaction: 1=not enough, 2=enough, 3=more than enough*

*^c^Frailty status: 1=robust, 2=pre-frail, 3=frail*

*CI: confidence interval*

**Table 3. Univariate logistic regression model to assess the association between sub-components of Frailty Index and SARS-CoV-2 infection—a sensitivity analysis**

|  | **Odds ratio** | ***p* value** | **95% CI** |
| --- | --- | --- | --- |
| Sum of all frailty components (0-30) | 1.11 | 0.061 | 1.00–1.23 |
| Chronic diseases (0-8) | 1.15 | 0.289 | 0.89–1.50 |
| Basic activities of daily livings (0-5) | 1.45 | 0.207 | 0.82–2.57 |
| Instrumental activities of daily livings (0-8) | 1.25 | 0.050 | 1.00–1.56 |
| Psychological symptoms and signs (0-6) | 1.03 | 0.849 | 0.76–1.40 |
| Self-reported health (0-1) | 3.86 | 0.077 | 0.86–17.3 |
| Physical measurement (0-1) | 1.63 | 0.226 | 0.74–3.60 |
| Lifestyle (0-1) | 0.81 | 0.647 | 0.33–1.98 |

*Notes: Numbers in the parentheses are the ranges of scores.*

*CI: confidence interval*
